# Three cases of corticosteroid therapy triggering ventricular fibrillation in J-wave syndromes

**DOI:** 10.1007/s00380-013-0443-x

**Published:** 2013-11-27

**Authors:** Naka Sakamoto, Nobuyuki Sato, Masahide Goto, Motoi Kobayashi, Naofumi Takehara, Toshiharu Takeuchi, Ahmed Karim Talib, Eitaro Sugiyama, Akiho Minoshima, Yasuko Tanabe, Kazumi Akasaka, Junichi Kawabe, Yuichiro Kawamura, Atsushi Doi, Naoyuki Hasebe

**Affiliations:** 1 Department of Cardiology, Asahikawa Medical University, Midorigaoka Higashi 2-1-1, Asahikawa, 078-8510 Japan; 2 Department of Cardiology, Asahikawa Red Cross Hospital, Asahikawa, Japan

**Keywords:** Corticosteroid therapy, J-wave syndrome, Prednisolone, Ventricular fibrillation

## Abstract

We describe three cases of J-wave syndrome in which ventricular fibrillation (VF) was probably induced by corticosteroid therapy. The patients involved were being treated with prednisolone for concomitant bronchial asthma. One of the three patients had only one episode of VF during her long follow-up period (14 years). Two patients had hypokalemia during their VF episodes. Corticosteroids have been shown to induce various types of arrhythmia and to modify cardiac potassium channels. We discuss the possible association between corticosteroid therapy and VF in J-wave syndrome based on the cases we have encountered.

## Introduction

Much attention has been focused recently on early repolarization and the so-called J-wave syndrome [1], because of the association between early repolarization patterns seen in electrocardiograms (ECGs) and the increased risk of idiopathic ventricular fibrillation (VF) [2, 3]. The triggering mechanisms underlying J-wave syndromes have not been fully elucidated. Regarding the causative genes for J-wave syndromes, various genetic mutations related to sodium, calcium, and potassium channels have been reported [1]. In addition, the triggering of VF is affected by factors such as the autonomic nervous system, hypokalemia, ischemia, febrile illnesses, and drugs, and gender differences and aging effects have been reported [1, 4]. However, to date the mechanisms and triggering factors responsible for VF in J-wave syndromes remain unknown.

Steroid therapy may induce various arrhythmias, including sinus bradycardia, supraventricular tachycardia, atrial fibrillation, and ventricular tachycardia [5, 6]. There has been one case report of VF related to hypokalemia induced by steroid therapy in a patient with Brugada syndrome [7]. Steroids cause hypokalemia via their mineralocorticoid action, so hypokalemia is likely a key trigger of VF in J-wave syndromes, as in the case reported [1, 7, 8]. We have reported a case of early repolarization syndrome related to severe hypokalemia in a 66-year-old man [8]. On the other hand, stress, which may induce the release of intrinsic corticosteroids, is also a likely key triggering factor for VF.

We have encountered three cases of J-wave syndrome likely triggered by corticosteroid therapy. Here we discuss the possible link between steroid therapy or stress and VF episodes in J-wave syndrome.

## Case reports

### Case 1

The clinical characteristics of this female patient are described elsewhere; she had an R367H SCN5A mutation [9, 10]. In brief, a 37-year-old Japanese woman was referred to our hospital for recurrent syncope caused by VF. She had been treated for bronchial asthma in our hospital before the VF episode. We had no laboratory data in the outpatient clinic before the VF episode; however, the data on admission for recurrent VF showed hypokalemia (2.7 mEq/l), which was likely caused by prednisolone (5 mg) treatment for the concomitant bronchial asthma.

**Fig. 1:**
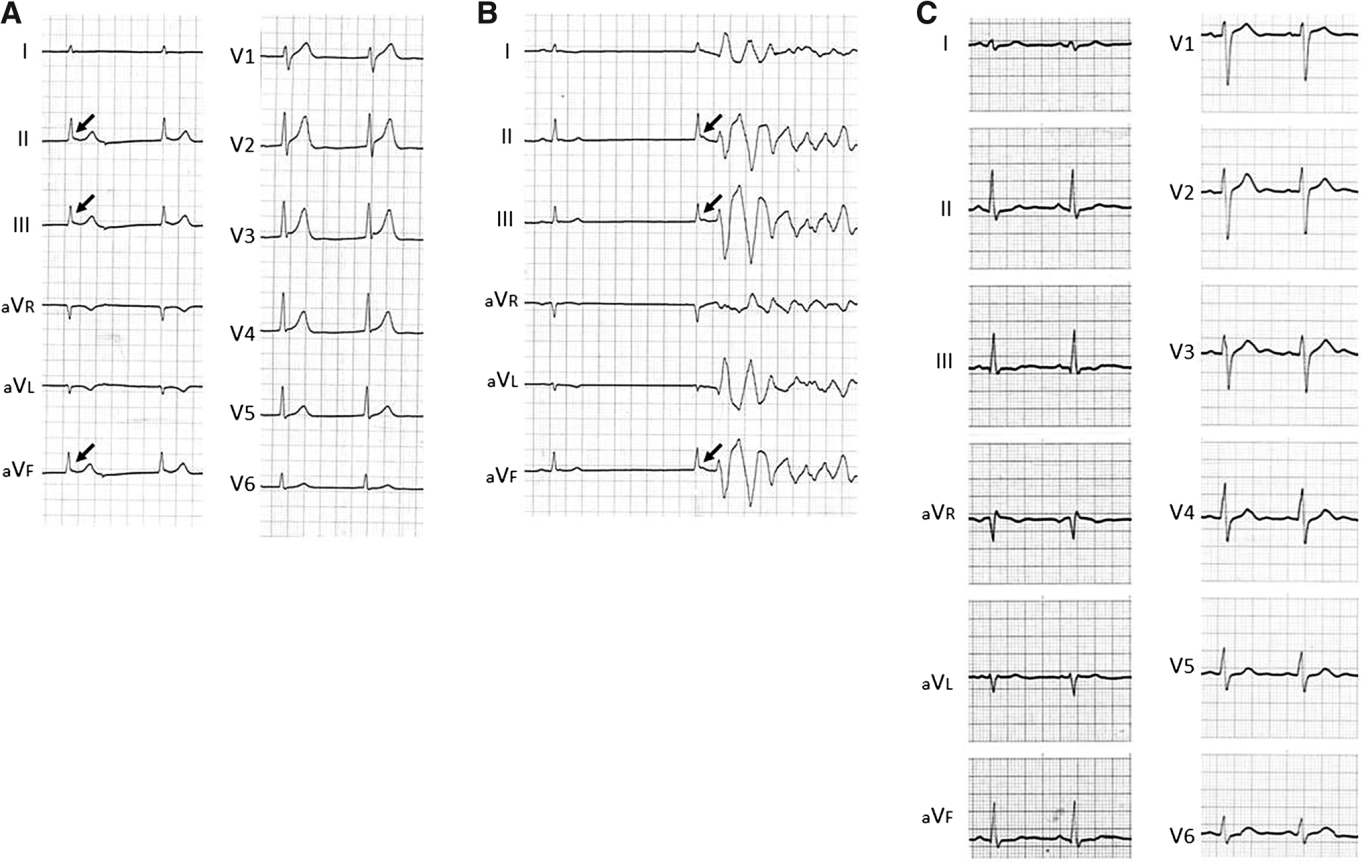
**a** Electrocardiogram (ECG) tracings obtained on admission during a ventricular fibrillation (VF) episode. Atrial standstill occurred in association with J-wave augmentation in leads II, III, and aVF (*arrows*). **b** Atrial standstill and a VF episode observed on admission. Note that the J waves in leads II, III and aVF were augmented after a long pause (*arrows*). **c** ECG obtained before the VF episode. Note the relatively wide S waves in leads I, II, III, aVL, and aVF, as well as the precordial leads, in addition to a slightly widened QRS. Also, neither a Brugada sign nor J-wave augmentation is present. The ECGs were modified from Takehara et al. [9], with permission

The ECG tracing on admission showed junctional rhythm with atrial standstill associated with J-wave augmentation in the inferior leads (Fig. 1a), and the J waves were augmented after a long pause, resulting in the development of VF (Fig. 1b). By contrast, in the ECG obtained before the VF episode, neither a Brugada sign nor J-wave augmentation was noted (Fig. 1c). The patient received an implantable cardioverter-defibrillator (ICD) and has experienced no VF episodes during a 14-year follow-up period. She was finally diagnosed as having early repolarization syndrome with SCN5A mutation [9, 10].

### Case 2

**Fig. 2:**
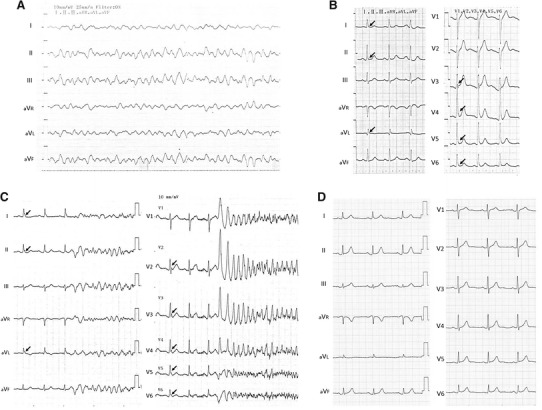
**a** Electrocardiogram (ECG) tracings during the first episode of ventricular fibrillation (VF). **b** ECG just after the VF episode. Note the J-wave augmentation in leads I, II, aVL, and V3–V6 (*arrows*). **c** ECG tracings during a VF storm. Note that the VF was triggered by a short coupled premature ventricular contraction and J-wave augmentation in leads I, II, aVL, and V2–V6 (*arrows*). **d** A recent ECG obtained during a VF-free period. The J-wave augmentation is less prominent

The patient’s detailed clinical profile is described elsewhere [11]. In brief, a 51-year-old man presented with his first VF episode during admission to the hospital for bronchial asthma therapy on April 15, 1998 (Fig. 2a). He had been treated with prednisolone (5 mg) and β-stimulator inhalation. Although cardiopulmonary resuscitation was successful, a complication involving ischemic encephalopathy occurred. The ECG just after resuscitation showed augmented J waves in leads I, II, aVL, and V3–V6, which were compatible with early repolarization syndrome (Fig. 2b). The patient received an ICD in 1999 and remained free from any VF episodes under medication with 450 mg mexiletine and 50 mg spironolactone.

In 2000, the patient experienced a repeat episode of VF. On readmission, a VF episode followed by an ICD intervention was documented on the ECG-monitor recordings. The recordings showed that the VF episode was triggered by a short coupled premature ventricular contraction with a coupling interval of 280 ms (Fig. 2c).

The patient has been free from any VF episodes while receiving combination therapy with mexiletine 300 mg, verapamil 120 mg, and spironolactone 50 mg for the past 13 years. A recent ECG obtained during a VF-free period is shown in Fig. 2d. No prominent J-wave augmentation has been seen since the second VF episode.

### Case 3

A 57-year-old man was found to be in distress in bed at midnight on October 28, 2008, so his family called for an ambulance. A VF episode was documented, and cardiopulmonary resuscitation and defibrillation were successful. The patient was then transferred to Asahikawa Red Cross Hospital, and hypothermia therapy was started. Laboratory data on admission showed hypokalemia (3.0 mEq/l). The patient was finally discharged without any neurologic deficits, and he was referred to our hospital for insertion of an ICD on November 5, 2008.

**Fig. 3:**
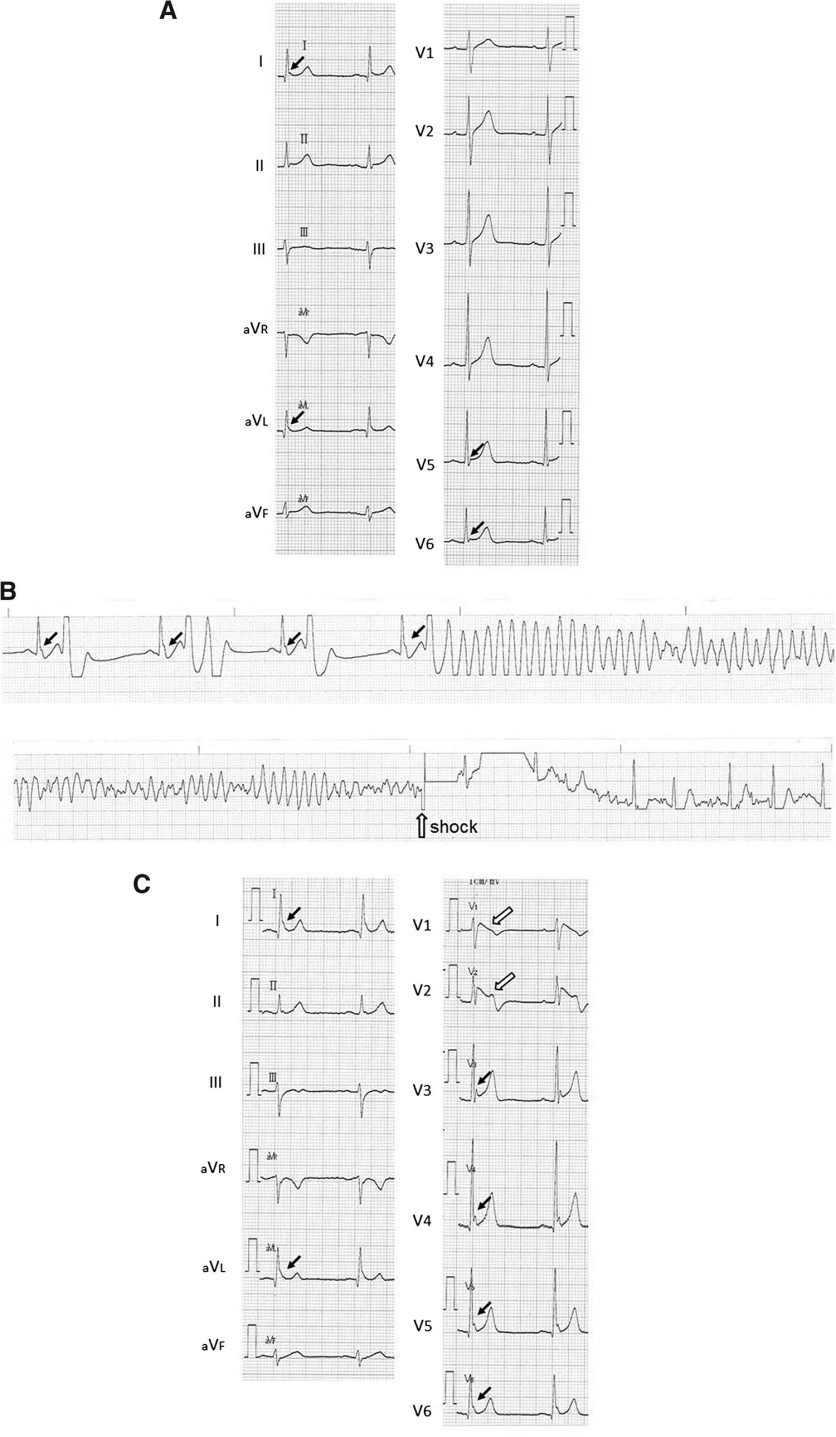
**a** Electrocardiogram (ECG) during the day in the absence of a ventricular fibrillation (VF) episode. Note the J waves in leads I, aVL, V5, and V6 (*arrows*); however, no Brugada sign is present. **b** ECG monitor strip recorded in an ambulance. The VF was initiated by a short coupled premature ventricular contraction. Prominent J-wave augmentation (*closed arrows*) is also noted. **c** ECG tracing during a VF storm. Note the coved-type ST elevation in leads V1 and V2 (*open arrows*) associated with J-wave augmentation in leads I, aVL, and V3–V6 (*closed arrows*)

The patient’s ECG showed J waves in the lateral leads, with no Brugada-type ST elevation (Fig. 3a). Clinical examination, including ultrasonography, pilsicainide challenge test, and cardiac catheterization, showed no particular abnormalities, so the patient was diagnosed with idiopathic VF. He was free from any VF episodes for 5 years after receiving the ICD. In addition, ECGs recorded in the outpatient clinic showed no Brugada-type findings or J-wave augmentation.

In January 2013, the patient presented with a chronic cough, and cough-variant asthma was diagnosed. He was treated with prednisolone 5 mg/day and β-stimulant tape. On April 2, 2013, during his routine ICD check, a VF episode followed by an ICD intervention was observed. The clinician thought that the β-stimulant tape may have caused the VF episode, so he advised the patient to stop using it. However, an electrical storm began the next day (Fig. 3b), so the patient was readmitted to our hospital. On admission, his laboratory data showed a tendency toward hypokalemia (3.6 mEq/l), probably caused by the prednisolone. The ECG tracing showed a coved-type ST elevation in leads V1 and V2 associated with J-wave augmentation in leads I, aVL, and V3–V6 (Fig. 3c). The electrical storm finally stopped after an isoproterenol infusion, discontinuation of prednisolone, and correction of the electrolyte imbalance. The patient has been free from any VF episodes since then. From these results and observations, the patient was finally diagnosed as having Brugada syndrome with inferolateral J waves.

## Discussion

In the present report we describe three cases of J-wave syndrome in which the patients experienced VF during corticosteroid therapy. We could not show any direct evidence for a link between the corticosteroid therapy and the arrhythmias; however, we consider the steroid therapy and subsequent hypokalemia to be related to the VF episodes in these patients with J-wave syndrome, because these very rare VF episodes each occurred in a patient receiving corticosteroid therapy.

Corticosteroid therapy, especially when associated with steroid pulse therapy, can induce both tachyarrhythmias and bradyarrhythmias [5]. From the basic electrophysiologic viewpoint, corticosteroids affect cardiac potassium and calcium channels [12, 13].

Fujimoto et al. [14] reported the effects of intravenous methylprednisolone pulse therapy on cardiac rhythm and electrolyte metabolism in 25 patients with nephrotic syndrome, and found that ventricular arrhythmias, including ventricular tachycardia, were induced in association with an increased fractional excretion of potassium. They proposed that potassium efflux from the cell caused by the effects of methylprednisolone on the cell membrane may lead to cardiac rhythm disturbances, and also speculated that the rate of change in the concentration of potassium, rather than its absolute concentration, seem to be important in the development of ventricular arrhythmias. In the cases reported here, two of the three patients had hypokalemia, suggesting that hypokalemia, probably induced by corticosteroid therapy, may be closely related to VF episodes, because it is generally accepted that glucocorticoids reduce serum potassium concentration via their mineralocorticoid action. Moreover, in the cases of VF storm we have encountered, in early repolarization syndrome the rapid change in potassium concentration may have been related to the patients’ lethal arrhythmias [8], which would be compatible with the findings of Fujimoto et al. [14].

To date, only one case of corticosteroid therapy-induced VF exacerbated by hypokalemia in Brugada syndrome has been reported [7]. Regarding the relation between corticosteroids and potassium channels, deoxycorticosterone acetate salt treatment alters the amount of K_v_ transcripts, suggesting that mineralocorticoids may be involved in K_v_ gene expression [15]. Chronic dexamethasone treatment also decreases the transient outward current (*I*_to_) density [16], which would be expected to enhance the transmural dispersion of repolarization. In Andersen–Tawil syndrome, a rare disorder of periodic paralysis caused by mutations in the KCNJ2 gene, which encodes the inward rectifier potassium channel Kir 2.1, stress or corticosteroids exacerbate the symptoms [17, 18]. Based on these clinical reports and cellular electrophysiology studies, we speculate that corticosteroids, through their effects on cardiac potassium channels and the resulting hypokalemia, may trigger VF in patients with J-wave syndrome.

In the three cases described here, two patients (cases 1 and 3) had hypokalemia or a tendency toward hypokalemia just before their VF episodes. Furthermore, in case 2 the patient had a relatively low potassium concentration (3.8 mEq/l) during his second VF episode, after which spironolactone was given to prevent further VF, and there was no recurrence. These observations suggest that the hypokalemia subsequent to corticosteroid therapy may be a critical factor in initiating VF episodes in patients with J-wave syndromes. On the other hand, as described above, steroids themselves have an arrhythmogenic effect by affecting potassium channels either directly or through modulation via secondary hypokalemia. The VF episodes occurred mainly with corticosteroid therapy during a long follow-up period, so a combined effect of steroids and secondary hypokalemia may be one of the triggering factors for VF in patients with J-wave syndromes.

Considering the cases presented here, we suggest that physicians consider the possibility of VF resulting from the use of corticosteroids and subsequent hypokalemia in patients with J-wave syndrome.

### Limitations

We present evidence for a possible link between corticosteroid therapy and VF episodes in patients with J-wave syndromes. VF may be caused by the direct or indirect effects of corticosteroid therapy on potassium channels. However, we have no direct evidence of an association between corticosteroid therapy and VF from the clinical and basic electrophysiologic viewpoints. Therefore, our discussion is based mainly on speculation. Further studies on the triggering factors for VF in J-wave syndromes, and the relation between the use of corticosteroids and VF, are needed to elucidate possible mechanisms.
